# Radiofrequency Energy in Hepatic Bed during Partial Cystectomy for Hydatid Liver Disease: Standing Out from the Usual Conservative Surgical Management

**DOI:** 10.1155/2016/1078653

**Published:** 2016-07-21

**Authors:** Eleftherios Mantonakis, Alexandros Papalampros, Demetrios Moris, Nikolaos Dimitrokallis, Panagiotis Sakarellos, John Griniatsos, Evangelos Felekouras

**Affiliations:** 1st Department of Surgery, Laikon General Hospital, Athens Medical School, National and Kapodistrian University of Athens, 11527 Athens, Greece

## Abstract

*Background*. Surgical treatment of hydatid liver disease (HLD) is divided into conservative and radical procedures. While conservative techniques are easier and faster to perform, there is an emerging need to reduce their morbidity and recurrence rates. Our aim was to present and evaluate the efficiency and safety of the application of radiofrequency energy (TissueLink® and Aquamantys® systems) in hepatic bed during partial cystectomy.* Materials and Methods*. Eighteen consecutive patients with hydatid liver cysts were referred to our department between April 2006 and June 2014. Data about demographics, mortality, morbidity, and recurrence rate were obtained and analyzed retrospectively.* Results*. The mean follow-up was 38 months (range: 4–84 months). The postoperative course of most patients was uneventful. One case of recurrence was found in our series in a patient with 4 cysts in the right lobe, 3 years after initial treatment. He was reoperated on with the same method.* Conclusions*. Saline-linked RF energy seems to be an effective means to be employed in conservative surgical procedures of HLD, with satisfactory postoperative morbidity. Recurrence rates appear to be low, but further follow-up is needed in order to draw safer conclusions.

## 1. Introduction

Hydatid liver disease (HLD) or echinococcosis is a zoonosis, caused by the tapeworm* Echinococcus*. The most commonly encountered cystic disease is caused by* E. granulosus* and is highly endemic in the Mediterranean countries (Spain, France, Greece, Turkey, Tunisia, etc.), the Middle East (Israel, Iran), South America (Argentina, Chile), and certain areas of Asia (China, India) [1–4]. With the observed population shifts because of migration and ease of travel in the modern era, echinococcosis has the potential of becoming a worldwide disease, creating a need for increased information and alert among physicians. It is estimated that 1.2 million people are affected and more than 1 million Disability Adjusted Life Years (DALYs) are lost globally [5].

The diagnosis of HLD often requires a combination of patient history, clinical examination, serology, and imaging. Imaging has been the mainstay of diagnosis, with ultrasonography playing an important role in the categorization of cysts. Gharbi et al. [6] first proposed a classification of CE based on ultrasonography findings in 1981, with WHO publishing an international classification in 2003 [7]. Since then, various improvements in the staging and scoring system have been proposed [8]. Computed tomography (CT) is an extremely helpful modality, especially in the planning of surgery. It provides more accurate information about the cyst location and the depth, while it clearly shows the presence of daughter cysts and exogenous cysts [9]. Other diagnostic tools like magnetic resonance imaging and cholangiopancreatography are employed under more specific indications [10].

Surgical treatment is still considered as the cornerstone of treatment despite the well-established role of medical treatment in the natural history of HLD. Almost all such surgical techniques have the common property of inactivation and evacuation of the cyst contents. The differences between the techniques consist of the removal of cyst cavity and rise controversies about the optimal operating technique [11]. The success of each approach lies in the ability to destroy nonvisible cyst remnants or exophytic growth, while sealing blood vessels and smaller bile ducts in the hepatic bed. Surgeons in endemic areas have accumulated extensive experience and reported large series of patients, documenting recurrence rates of 7.7%–30% and morbidity of 21%–80% with conservative surgical therapies [12, 13].

The role of radiofrequency (RF) energy systems in liver surgery and especially in HLD is already studied with encouraging results as far as recurrence, safety, and feasibility are concerned [14, 15].

In this retrospective study, we present a novel surgical technique for the treatment of hydatid liver disease by applying RF systems (TissueLink and Aquamantys) in hepatic bed during partial cystectomy approach. To the best of our knowledge, this is the first application of saline-linked RF energy in the literature for the treatment of HLD.

## 2. Materials and Methods

We retrospectively analyzed data from 18 consecutive patients with HLD who were referred and operated on in our department between April 2006 and June 2014. In order to identify study patients, we searched discharge abstracts for diagnoses of hydatid disease based on the International Classification of Diseases- (ICD-) 10 (codes 122.8 and 122.9) and ICD-9 (codes B67.8 and B67.9). Individual surgeon records were also mined for any missing cases. We retrieved information and data about the age, the gender, the presenting symptoms of the patients, the number of cysts, its location, and its classification according to WHO criteria [7]. Informed consent was given from all patients and IRB approval was obtained. Postoperative complications were evaluated by Dindo-Clavien classification [16].

All patients underwent hydatid serology (with two different techniques including enzyme-linked immunosorbent assay [ELISA], hemagglutination, and indirect immunofluorescence assay) and imaging assessment of liver hydatid disease by abdominal ultrasound, computed tomography (CT) scan, or magnetic resonance imaging (MRI) [10]. Chest CT or X-rays were systematically performed to identify associated pulmonary hydatid cyst. Endoscopic retrograde cholangiopancreatography (ERCP) was performed in cases with evidence of large biliocystic fistula with migrated endobiliary hydatid vesicles.

The therapeutic algorithm of our center includes partial cystectomy assisted by saline-linked RF energy in order to destroy nonvisible cyst remnants or exophytic growth, while sealing blood vessels and smaller bile ducts. The TissueLink monopolar sealer was used in 8 patients from 2006 to 2010 and its successor, the Aquamantys® bipolar sealer in 10 patients from 2011 to 2014.

### 2.1. The Instruments

The TissueLink dissecting sealer is a RF energy coagulator, which employs RF energy and conducts it through saline irrigation into the target tissue where it is converted into heat. The saline facilitates transfer, while it serves as an even distributor of energy on the target tissue, but also acts as a coolant, since its continuous flow does not allow the temperature to rise above 100°C. In this way, there is tissue coagulation and collagen shrinkage and obliteration of small vessels and bile ducts but no charring and scar formation [17]. Small vessels and bile ducts up to 3 mm in diameter are sealed efficiently using the dissecting sealer and there is no need to be ligated [18, 19]. The TissueLink needed to be connected with a compatible standard electrocautery generator. The newer Aquamantys system uses the same operating principles, with the difference that it is bipolar, so its application is faster and safer for the patient, while it has its own generator device. These devices are known to disrupt the extracellular target matrix with a coagulative band in a distance of 2 mm from the application margin [19].

### 2.2. Our Technique

**Figure 1:**
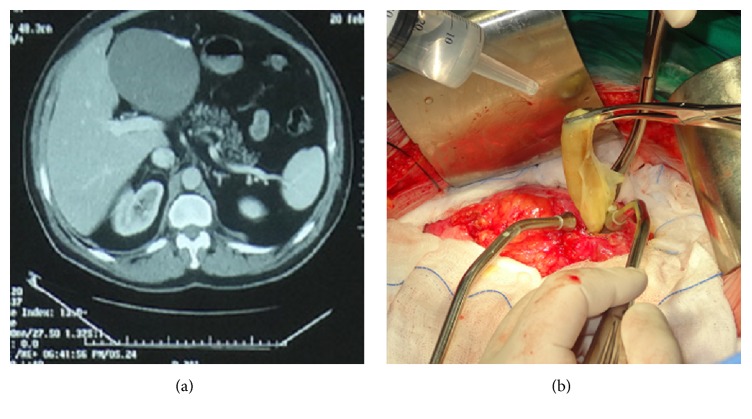
(a) Preoperative CT of a patient with a large left lobe hydatid cyst, obliterating the LPV and in contact with the RPV. (b) Intraoperative photo of the endocyst removal, after aspirating and opening the cyst.

**Figure 2:**
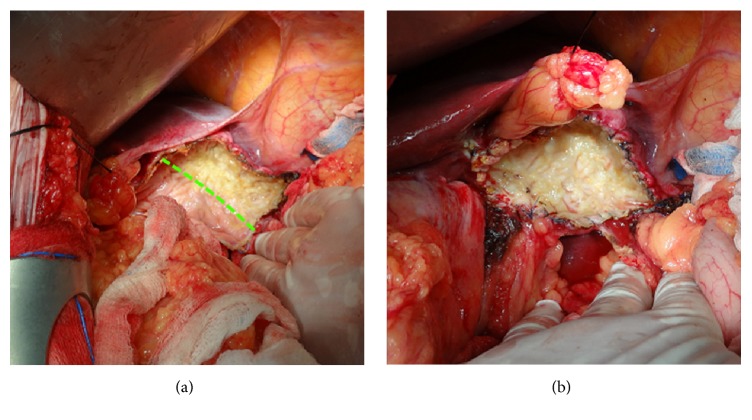
Operative field after (a) partial ablation of the cyst's bed with the Aquamantys system (yellow top right above the green line, in contrast to the nonablated pink lower left below the green line) and (b) complete ablation of the cyst bed with the Aquamantys system and oversewing the cyst's edges to avoid bleeding and bile leakage.

As we have already described [20], we approach HLD with an extended right subcostal incision that can be extended to the left, as a bisubcostal incision, according to the topography and number of cysts and cysts are identified with intraoperative ultrasound to ascertain their exact extent and relation with major structures. Pads soaked in 15% hypertonic solution (used as scolicidal agent) are used for protection of the operating field from contents spillage and contamination. After opening the cyst, a large bore trocar was used to aspirate its content and cyst cavity is filled with 15% hypertonic solution for 10 minutes and reaspirated. A second suction device is often useful to avoid spillover contamination of the abdominal cavity should any fluid escape around the trocar. Cystectomy is performed by working in the cleavage plan between endocyst and pericyst and by dissecting as much of the endocyst off the pericyst as possible (Figures 1 and 2). Drainage at the bottom of the cavity is routinely performed.

In cases that vessels are close to the cyst to treat, the technique above seems to be safe and feasible since we first perform cystectomy and after that we apply the Aquamantys on the fibrous capsule. Fibrous capsule protects biliary architecture and vasculature exposure to the RF energy. Since we operate for benign disease, there is no reason to be aggressive and expose the liver parenchyma and the vessels. Moreover, the tip of Aquamantys device is nontraumatic. Finally, we anchor the cyst remnants on liver parenchyma that facilitates us to avoid bile leak.

## 3. Results

The median age in our series was 49 years (range: 32–71). Around 83% of the patients were men and only 3 cases were noted in women. The mean follow-up was 38 months (range: 4–84 months). No patient was lost during the follow-up period.

Three of the patients had fever as the presenting symptom, 1 had localized right upper quadrant pain, and 5 had nonspecific abdominal symptoms, while the rest were accidentally diagnosed, after an imaging study performed for another reason. Three of the patients had been previously operated on for HLD with another method. Sixteen patients had single cysts and two multiple. Ten out of 18 were located in the right lobe, 5 in the left, 2 bilateral, and 1 in the caudate lobe (Table 1). The majority of patients (61%) were preoperatively classified with ultrasound as type CE3 or CE2 according to WHO criteria [7], whereas no case of CE1 was found in our series. Seven patients who were classified as CE4 underwent the operation due to presence of symptoms. All patients were treated preoperatively with albendazole (10 mg/kg/day) for 2 weeks which was continued postoperatively for 4 weeks. The median duration of surgery was 195 (range 145–395) minutes.

The postoperative course of most patients (83.3%) was uneventful (no Dindo-Clavien classified complication). The median length of stay in hospital was 9 (range: 6–18) days. One patient (5.6%) with a large cyst of sectors VI, VII, VIII, and IV developed a low output bile fistula, which resolved after 10 days of conservative treatment. One patient (5.6%) with a large cyst of the right lobe in contact with the diaphragm developed a right pleural effusion, which was with percutaneous drainage. One of the patients (5.6%) who presented with recurrence was operated on with 4 cysts of the right lobe and recurred in 1 of the cysts after 3 years. He was reoperated on with the same method. None of our patients needed transfusion during the postoperative period. No patient died during the follow-up period.

Our aim by using these instruments was to apply saline-linked RF energy in order to seal any open bile ducts or blood vessels while at the same time coagulate in sufficient depth so that no exogenous cysts are viable. Furthermore, in difficult cases where parts of the cysts were in close proximity to great vessels, RF energy facilitated us to destroy parts of the endocyst.

**Figure 3:**
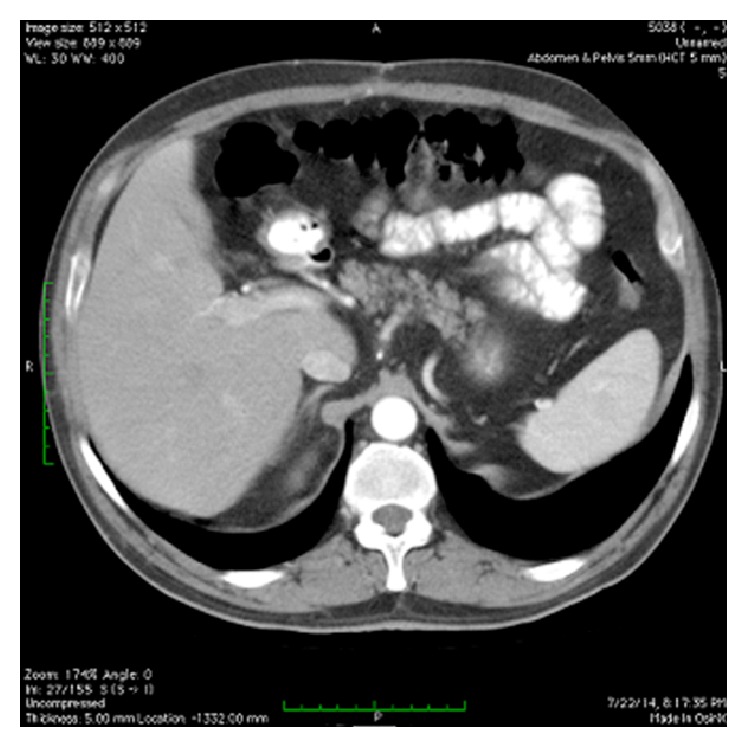
Postoperative CT of the same patient.

In six cases, upon opening the cysts, a bilus hue of the fluid was observed and was followed by a meticulous search for a visible bile duct. In only two cases was the duct identified and sutured with 3-0 prolene sutures. The TissueLink/Aquamantys systems were used in order to ablate the entire pericyst surface with the purpose of providing hemostasis and seal nonvisible bile ducts, while destroying any possible exophytic extension of the endocyst. In two cases (cyst in caudate lobe and one in the right lobe) a part of the pericyst was in close relation to great vessels (inferior vena cava; IVC, right portal vein; RPV) and we judged that performing total cystectomy would lead to increased operative risk. Therefore, a great portion of the pericyst was removed, while its remnants close to the vessels were destroyed with the use of saline-linked RF energy. The intraoperative result was satisfactory in all cases and no further measures were needed (Figures 2 and 3).

**Figure 4:**
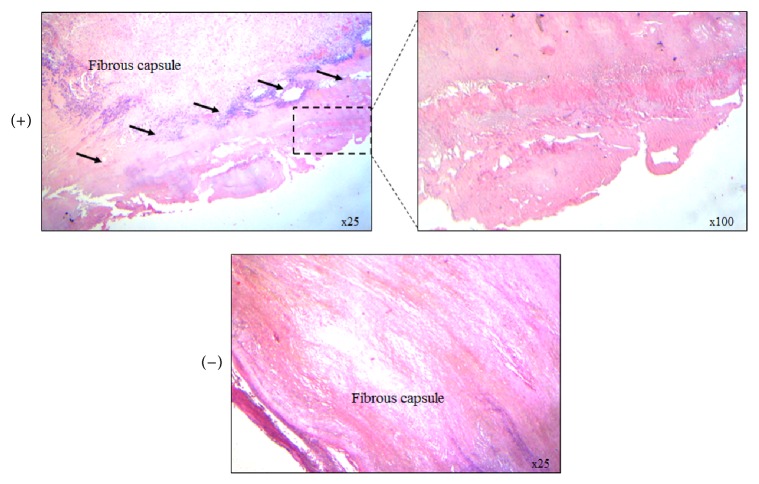
Histology section of a single patient: RF ablation induces coagulative necrosis in the cyst wall of* Echinococcus granulosus* hydatid cyst. Arrows denote area of coagulative necrosis.

Histology section of a single patient confirmed that RF ablation induces coagulative necrosis in the cyst wall without parasite activity (Figure 4) that could be the crucial pathological step of the success of the procedure.

## 4. Discussion

Surgery remains the mainstay of treatment of HLD, especially in complicated cases (rupture, pressure of vital organs, infection, hemorrhage, and communication with biliary tree). The four critical goals of surgical treatment are the neutralization of all infectious material, the evacuation of the cyst cavity, while taking severe measures to avoid spillage of its contents, and the obliteration of the residual cavity. Perioperative chemoprophylaxis with BMZ, starting 1 week before surgery and until the fourth postoperative week, should be administered to reduce the risk of secondary echinococcosis from intraoperative spillage of cyst contents [10].

RF energy has been previously used in small series of patients most often in percutaneous applications as a scolicidal agent (in the ablation setting) or as an intraoperative tool to perform surgical procedures like pericystectomy and cystectomy. More specifically, Brunetti and Filice [21] used RF in a percutaneous ablation setting in two patients with large Gharbi IV cysts, previously treated for 2 years with albendazole. One patient had a 10 cm cyst in the right liver and the other a 10 cm in the right liver and a 12 cm cyst in the middle liver. They used a 14 G outer needle, housing ten solid retractable curved electrodes, which when deployed, looked like an umbrella of 3.5 cm in diameter. The cysts were ablated in a similar pattern used for solid tumors, like hepatic metastases. Histologic examination of the material removed with suction after the ablation showed no parasite activity. None of the two patients showed complications or recurrence of the disease. As we have already mentioned, histologic confirmation of no parasite activity and presence of coagulative necrosis are important findings, indicative of an encouraging prognosis as far as recurrence is concerned. In the same frame, Bastid et al. [22] reported the percutaneous treatment of a complex hydatid cyst, using RF in the ablation setting under ultrasound control, along with albendazole therapy. After the ablation, alcohol was injected in order to induce retraction of the residual cavity. No complications were reported, while after six months the patients had no signs of recurrence. The six-month follow-up period of the specific patient has been evaluated as being insufficient by some authors [23], since the patient had a CE2 cysts with primary liquid component and their experience with similar patients was rather disappointing after longer follow-up.

Papaconstantinou et al. [14] in a previous study from our center reported the intraoperative use of RF energy in 3 patients subjected to total pericystectomy with the open method. After emptying and neutralizing the cyst, the needle electrode was inserted within the hepatic parenchyma adjacent to the cyst and RF energy was applied at 160 W/1 A in consecutive sites in order to create an ablated tissue zone around the cystic cavity. The complete ablation of a site was followed by sharp division of the parenchyma. When vascular structures and bile ducts were encountered, meticulous RF energy was applied. There were no complications or recurrence after 2 years of follow-up. The authors noted that RF energy, despite its efficiency in select cases, is less efficient in sealing major vessels, while it can cause unwanted thrombosis and thermal injury to bile ducts or vessels to be preserved. It is important that RF energy as a tool in radical procedures is used by experienced liver surgeons.

Sahin et al. [15] also reported their experience with two patients with HLD, treated with cystectomy and pericystectomy with the intraoperative use of RF energy. As we also demonstrate in our series, no transfusions were reported during the operation. Similarly, Giorgio et al. [24] performed RF, in the ablation setting, with an expandable needle in 5 patients with CE4 disease, which were still viable after PAIR. Three to six months after the procedure no viable scolices were detected, while in 4 out of 5 patients the cysts showed a 60% decrease in size.

More recently in an ex vivo experimental animal study, Lamonaca et al. [25] used RF with an expendable umbrella-like needle electrode in the ablation setting, for the treatment of 9 liver and 7 lung cysts from 12 slaughtered animals. After ablation the cysts were removed and pathology showed 100% success rate in all cysts and an immediate volume reduction of at least 65%. An important observation was that the endocyst was still attached to the pericyst in the great majority of cases, a finding unlike what is commonly observed after PAIR (puncture, aspiration, injection, and reaspiration) and pericystectomy surgery. This could imply that the method, when used in vivo, could lead to less biliary fistula formation and postoperative complications.

From the aforementioned data it is evident that while conservative techniques are easier and faster, there is an emerging need to reduce their morbidity and recurrence rates. The application of modern technology could assist in that direction. While RF technology has been used mainly in the ablation setting or as a tool in hepatectomies, it has not been used to be the best of our knowledge in conservative procedures. Results can be thought as satisfactory, as far as complications related to surgical technique are concerned; in our small series of patients only one low output bile fistula was observed and it spontaneously resolved. Only one recurrence in a patient with 4 right lobe cysts, who had previously been reoperated on, was observed after 3 years.

The efficacy and safety of our technique seem reasonable when compared with studies of similar design and demographics [26] where the mortality rate reached 2.76%, postoperative complication rate was 24.13%, and recurrence rate was 6.9%. In that study, no RF energy device was used and the majority of patients underwent radical surgical treatment. Similarly, a recent study from a Western Center demonstrated far higher rates of morbidity (48%) and recurrence (10%) in the group of patients receiving conservative surgical treatment [27]. Recently, a retrospective study demonstrated a high rate of postoperative morbidity (36.5%) and bile leakage (14.8%) in patients with HLD treated with conservative treatment without RF energy devices [28]. Finally, a retrospective study, with similar number of patients to our series, showed a morbidity rate of 17.8% in the group of conservative surgical treatment and only one case of bile leak (3.6%) without the use of RF devices [29].

All in all, the rationale for the use of such a technique for the management of HLD lies in the fact that this technique seems to be efficient in terms of postoperative mortality, morbidity, and cost. Moreover, it seems to achieve comparable to radical surgery results in terms of recurrence, in hands of experienced liver surgeons [30, 31]. Of course, this technique is not a typical “conservative” one, since expertise in using RF-energy devices is needed and it is not always accessible in general nonexpert centers in endemic areas.

This study has several strengths. This is one of the very few studies evaluating conservative surgical treatment with the use of RF energy devices as a standard treatment approach for HLD, with results in terms of mortality, morbidity, and recurrence rates that are at least noninferior compared with studies of similar design for the same approach. This study though has several limitations. First, it was a retrospective review of medical charts and surgeon records. As with all retrospective series, there is a risk of bias related to the information quality available in medical records as well as its extraction. Although it is possible that additional cases of disease recurrence may have occurred among these patients, we argue that this is unlikely given the specialized nature of therapy for HLD and the likelihood that these patients would have been referred back to our center had recurrence occurred. Second, a low number of patients were included in our study, so conclusions should be carefully drawn. Finally, the noncomparative design of the study does not facilitate the recommendation according to efficiency of this device.

## 5. Conclusions

Saline-linked RF energy can be an effective means to be employed in conservative surgical procedures of HLD, with satisfactory postoperative morbidity in our series of 18 patients. Recurrence rates appear to be low, but prospective studies are needed to establish the superiority of this approach in treating HLD.

## Figures and Tables

**Table 1:** Patients' characteristics.

*Presenting symptoms *(*N* = 18)	
Nonspecific	6 (33.3%)
Fever	3 (16.6%)
Pain	1 (5.6%)
Accidental finding	5 (27.8%)
Follow-up (recurrence)	3 (16.7%)

*Number of cysts *(*N* = 18)	
Single cyst	16 (88.9%)
Multiple cysts	2 (11.1%)

*Location of cysts *(*N* = 18)	
Right lobe	10 (55.6%)
Left lobe	5 (27.7%)
Bilateral	2 (11.1%)
Caudate lobe	1 (5.6%)

*WHO cyst classification *(*N* = 18)	
Type CL	
Type CE1	0 (0%)
Type CE3a	2 (11%)
Type CE2/3b	9 (50%)
Type CE4	7 (39%)
Type CE5	0 (0%)
